# SNP barcodes provide higher resolution than microsatellite markers to measure *Plasmodium vivax* population genetics

**DOI:** 10.1186/s12936-020-03440-0

**Published:** 2020-10-20

**Authors:** Abebe A. Fola, Eline Kattenberg, Zahra Razook, Dulcie Lautu-Gumal, Stuart Lee, Somya Mehra, Melanie Bahlo, James Kazura, Leanne J. Robinson, Moses Laman, Ivo Mueller, Alyssa E. Barry

**Affiliations:** 1 Population Health and Immunity Division, The Walter and Eliza Hall Institute of Medical Research, Melbourne, VIC Australia; 2 Department of Medical Biology, The University of Melbourne, Melbourne, VIC Australia; 3 Vector Borne Diseases Unit, Papua New Guinea Institute of Medical Research, Madang, Papua New Guinea; 4 Malariology Unit, Institute of Tropical Medicine, Antwerp, Belgium; 5 Disease Elimination Program, Burnet Institute, Melbourne, VIC Australia; 6 Centre for Global Health and Diseases, Case Western Reserve University, Cleveland, Ohio USA; 7 Department of Parasites and Insect Vectors, Institut Pasteur, Paris, France; 8 Present Address: IMPACT Institute for Innovation in Mental and Physical Health and Clinical Translation, Deakin University, 75 Pigdons Road, Waurn Ponds, Geelong, VIC 3216 Australia; 9 Present Address: Department of Biological Sciences, Purdue University, West Lafayette, Indiana USA

**Keywords:** Malaria, *Plasmodium vivax*, Microsatellites, Single Nucleotide Polymorphisms (SNPs), Diversity, Population structure, Papua New Guinea

## Abstract

**Background:**

Genomic surveillance of malaria parasite populations has the potential to inform control strategies and to monitor the impact of interventions. Barcodes comprising large numbers of single nucleotide polymorphism (SNP) markers are accurate and efficient genotyping tools, however may need to be tailored to specific malaria transmission settings, since ‘universal’ barcodes can lack resolution at the local scale. A SNP barcode was developed that captures the diversity and structure of *Plasmodium vivax* populations of Papua New Guinea (PNG) for research and surveillance.

**Methods:**

Using 20 high-quality *P. vivax* genome sequences from PNG, a total of 178 evenly spaced neutral SNPs were selected for development of an amplicon sequencing assay combining a series of multiplex PCRs and sequencing on the Illumina MiSeq platform. For initial testing, 20 SNPs were amplified in a small number of mono- and polyclonal *P. vivax* infections. The full barcode was then validated by genotyping and population genetic analyses of 94 *P. vivax* isolates collected between 2012 and 2014 from four distinct catchment areas on the highly endemic north coast of PNG. Diversity and population structure determined from the SNP barcode data was then benchmarked against that of ten microsatellite markers used in previous population genetics studies.

**Results:**

From a total of 28,934,460 reads generated from the MiSeq Illumina run, 87% mapped to the *PvSalI* reference genome with deep coverage (median = 563, range 56–7586) per locus across genotyped samples. Of 178 SNPs assayed, 146 produced high-quality genotypes (minimum coverage = 56X) in more than 85% of *P. vivax* isolates. No amplification bias was introduced due to either polyclonal infection or whole genome amplification (WGA) of samples before genotyping. Compared to the microsatellite panels, the SNP barcode revealed greater variability in genetic diversity between populations and geographical population structure. The SNP barcode also enabled assignment of genotypes according to their geographic origins with a significant association between genetic distance and geographic distance at the sub-provincial level.

**Conclusions:**

High-throughput SNP barcoding can be used to map variation of malaria transmission dynamics at sub-national resolution. The low cost per sample and genotyping strategy makes the transfer of this technology to field settings highly feasible.

## Background

*Plasmodium vivax* is the most widely distributed human malaria parasite outside sub-Saharan Africa, accounting for approximately 7.4 million clinical cases per year [1]. Despite previously being categorized as a benign infection, studies have revealed that *P. vivax* can cause severe and life-threatening malaria and some cases may be drug resistant [2–5]. Features of *P. vivax* biology such as relapse, low-density infections and the appearance of transmission forms (gametocytes) prior to detectable clinical symptoms [6, 7], are a challenge for controlling and eliminating this disease. These characteristics, in addition to the high proportion of asymptomatic *P. vivax* infections in combination with increasing human movement also pose a significant challenge to malaria elimination [8, 9]. Establishing a strong malaria surveillance system is essential to monitor the changing malaria landscape and to achieve elimination goals.

Currently endemic countries rely on traditional malaria surveillance approaches such as Light Microscopy (LM) and rapid diagnostic test (RDTs) [10, 11], or more recently, through molecular diagnosis (PCR) of infection [12] to measure infection prevalence and provide an estimate of transmission intensity. Population genetics however can measure parasite genetic diversity, population structure, gene flow and relatedness of genotypes to help define transmission “zones”, local transmission dynamics [13, 14], to identify the source(s) of outbreaks [15], distinguish between local or imported cases [16, 17], and track imported infections [18–20]. In combination with epidemiological data this can help to guide control strategies and to monitor the effect of interventions facilitate malaria elimination [21, 22].

Capturing accurate population genetic signatures and tailoring molecular tools to the local malaria transmission scenario depends on the appropriate selection and use of informative molecular markers [23]. For over a decade, malaria population genetists have used panels of 6-12 microsatellites for assessment of malaria parasite transmission dynamics, its origins and dispersal [24–26]. Microsatellites have several advantages including being neutrally evolving [27, 28], multiplexing can be easily done for ten or more markers in a single PCR cocktail and they are abundant in the genome [29]. Despite these advantages, microsatellites have high PCR amplification biases that may cause incorrect classification of dominant and minor haplotypes [30]. Furthermore the small number of markers, high mutation rate and difficulty of scoring alleles accurately decreases the resolution of microsatellite markers to identify related parasites [26]. Due to their high diversity, parasite population sub-structure may be missed in areas of high transmission since existing panels of 10 to 14 markers may provide inaccurate estimates of relatedness. Furthermore, microsatellite genotyping is difficult to standardize across laboratories, reproducibility is lacking, and amplicons require fragment analysis at core sequencing facilities [28, 31]. Whole genome sequencing (WGS) is not widely used in malaria-endemic countries since it is costly and data analysis needs advanced bioinformatic tools and expertise [18].

As countries intensify their control programmes and approach malaria elimination, a robust, cost effective, rapid and easy-to-use set of molecular markers is urgently required as an alternative genotyping tool to rapidly track disease spread and imported cases. So-called ‘barcodes’ composed of a panel of single nucleotide polymorphisms (SNPs) can be used to profile each parasite isolate and to generate population genetic insights [32–34]. SNP barcoding is more easily standardized across studies and offers more rapid and highly automated genotyping options compared to microsatellites [23, 35]. Putatively universal (global) barcodes of 42 *P. vivax* SNPs have been developed and tested for their utility to determine parasite population structure [33]. However, there has been limited validation of these markers in different endemic settings to determine whether they are informative for all *P. vivax* populations. Informative barcodes need to distinguish between populations circulating in distinct geographic areas at sub-national resolution, as this is essential to inform malaria control programmes.

Universal barcodes may not provide an accurate estimate of local population structure due to ascertainment bias occuring if the SNP panel used was developed for populations other than those to be studied. That is, SNPs may be polymorphic (informative) in some populations, but not in others [31, 36, 37]. Ascertainment bias is a limitation to measuring the true allele frequency [38] resulting in minor allele frequency (MAF) biases in populations not included in ascertainment group [38, 39]. Thus, global *P. vivax* SNP marker selection [33] may limit the resolution of this barcode to genotype parasite populations not closely related to the ascertainment group. Moreover, recent intensive malaria control activities may lead to changes in MAF with a loss of rare variants [19, 20] that could reduce the power of the existing SNP barcode to distinguish between different genotypes. The use of population genomic data to aid malaria control relies on having this insight at regional or sub-national resolution, and will vary for different endemic settings and stages in the elimination pipeline [23]. Therefore, validating available barcodes or developing a new barcode that accurately captures the diversity of a country’s parasite population will facilitate characterization of the local malaria transmission scenario.

Here, we describe the development of a SNP barcode designed to capture the diversity of *P. vivax* populations of Papua New Guinea (PNG), which has the highest transmission of *P. vivax* in the world [40, 41]. SNP barcodes have been employed in several studies in recent years [15, 16, 23, 33], however their performance has not been compared to microsatellites, a commonly used genotyping tool [42–44] and used in our previous studies [9, 45–47]. We thus compared parasite population genetics using the newly developed SNP barcode with that for microsatellite markers to address the following research questions: (1) do these two marker panels capture parasite diversity at the sub-provincial scale at the same resolution, and (2) which marker panel has higher resolution to capture parasite geographic connectedness and population structure? Comparisons of SNP and microsatellite data demonstrates the superiority of this locally validated SNP barcode for monitoring parasite populations and tracking the source of infections.

## Methods

**Fig. 1:**
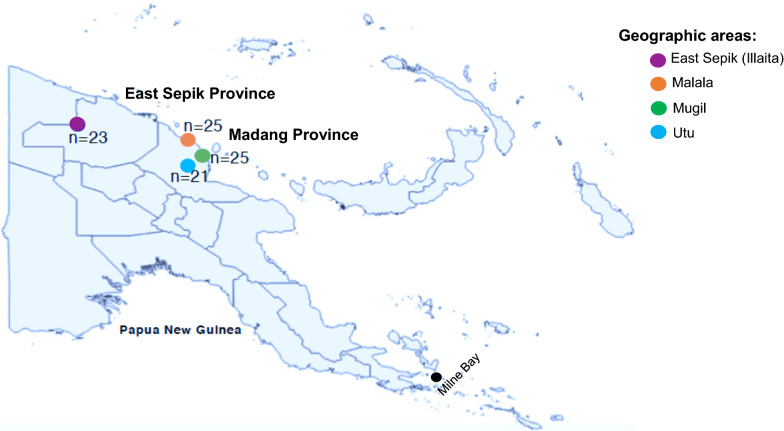
Map of the study area. Map of Papua New Guinea showing the location of four distinct catchment areas from which *P. vivax* isolates were obtained (colored dots). A total of 94 *P. vivax* isolates were selected from 2012/13 East Sepik and 2014 Madang cross-sectional surveys. Samples were genotyped using both SNP barcodes and microsatellites. n = the number of genotyped *P. vivax* isolates. Black dots indicate three provinces of PNG where 20 WGS *P. vivax* obtained for the assay development

This study aimed to develop a SNP barcode for high-resolution genomic surveillance of *P. vivax* in PNG (potentially applicable to other *P. vivax* endemic areas) and to benchmark it against an existing microsatellite marker panel that has been used in previous population genetic surveys. The study was performed using *P. vivax* isolates from four locations within a contiguous highly endemic region of the north coast of PNG, specifically in East Sepik (n = 1) and Madang Provinces (n = 3) (Fig. 1), where cross-sectional surveys and population genetic analyses were previously conducted using microsatellite markers [47]. Previous microsatellite analysis of these populations in 2005 and 2006 failed to identify any geographic population structure [46, 47].

### *Plasmodium vivax* isolates

For the genotyping, we selected a total of 94 *P. vivax* positive isolates collected during two cross-sectional surveys on the north coast of PNG including one population from East Sepik, collected in 2012/13 and three populations from Madang Provinces (Malala, Mugil and Utu) collected in 2014 (Fig. 1) [48]. These *P. vivax* isolates were selected due to the availability of published microsatellite data [49], which was used to compare with SNP barcode data.

### Selecting candidate SNPs

The software package, *Genome Analysis Toolkit* (GATK) [50] was used for selection of informative SNPs from published WGS data for 40 *P. vivax* isolates from three regions of PNG (Madang, East Sepik/Maprik and Milne Bay/Alotau) [14, 51] (Additional file 1: Table S1). WGS data quality was checked using *Fastqc* and coverage checked using *Bam coverage* to identify high quality genomes. To identify informative SNP variants, paired-end raw reads were aligned *to P*. *vivax* Salvador I strain (*PvSal1*) [52] using the *bwa*-*mem* mapping algorithm [53]. SNPs were called and filtered using *GATK HaplotypeCaller* [50]. To ensure uniform coverage of the whole parasite genome, variants present on 14 nuclear chromosomes were included after excluding all indel calls and ‘blacklisted’ highly polymorphic regions including the telomeres. Additional ‘hard filtering’ included retaining only biallelic single nucleotide variants (SNVs), high coverage (at least 90% of their bases covered up to 5x) SNPs, with a minor allele frequency (MAF) > 10% (0.10), with pairwise LD < 0.2 throughout a window of (0.5 kb), low positive or negative *Tajima’s D* values (|Tajima’s D| < 0.5) throughout a window of 0.5 kb, high heterozygosity (> 0.4) within PNG, and if they were relatively uniformly spaced across the *P. vivax* genome. From a total of 24,283 SNPs with MAF > 10%, 4006 remained after filtering and 220 relatively evenly spaced SNPs were selected for assay development (Additional file 2: Figure S1).

### SNP barcoding assay development

The assay consists of a series of 20 × 8-10-plex PCRs, with multiplexed amplification of the target regions (PCR#1) using Locus-Specific Primers (LSP) containing universal Illumina overhang adaptors (OH), attaching to all amplicons from each sample (PCR#2) a short sequence tag (multiplex identifier, MID) unique to each sample. This was followed by pooling of all amplicons after indexing each sample and sequencing on an Illumina MiSeq (Additional file 2: Figure S2).

The major problem with multiplex PCR is primer dimer formation and melting temperature (T_m_) variation between primers. To minimize these challenges, *PrimerPlex* software [54] was used to design multiplex PCRs using LSP pools for target 400bp genomic regions which contain SNPs of interest. A total of 22 multiplex PCRs were designed, with each containing 8–12 LSP pairs (220 SNPs total).

Primary multiplex PCRs were performed and optimized for each pool using published guidelines [55]. From a total of 220 SNPs, 42 were negative and were not amplified in the primary multiplex PCR. The remaining 178 SNPs were used for the assay development (Table S2). The optimized conditions for the primary PCR (PCR#1) required 2 μl of sample (20–40 ng DNA template) in a 20 μl reaction consisting of 0.3 mM each dNTP, 3 mM MgCl_2_, 1X buffer (B1), 0.2 μM primer pool, and 1unit of Hotstart DNA polymerase. The PCR conditions consisted of an initial denaturation step of 12 min at 95 °C, followed by 30 cycles of 15 s at 95 °C, 30 s at 60 °C, and 30 s at 72 °C, and a final 5 min extension at 72 °C.

### SNP barcoding assay optimization

To optimize the assay, a total of six (three monoclonal and three polyclonal infections) *P. vivax* positive field samples were genotyped using 20 randomly selected SNP markers (2 multiplex sets) from a total of 178 described in the above section. The amplicon sequencing approach was used to amplify all 20 *P. vivax* genomic loci with target SNPs in multiple samples at a time. Following the optimization, the assay was applied to a total of 94 *P. vivax* positive field samples.

Due to the small size of the *Plasmodium* genome in comparison to the human genome, the presence of just a few nucleated human cells in field specimens can impede genotyping or sequencing sensitivity and specificity by contributing a large proportion of unwanted human DNA to the DNA sample. Thus, to obtain enough parasite DNA from field samples and to minimize contaminating human DNA, digestion with the *Mcr*BC enzyme, which is a DNA methylation-dependent restriction enzyme (MDRE), followed by random whole genome amplification (rWGA) [56, 57] was used. In brief, digestion of human gDNA was done using the *Mcr*BC (methylation dependent) enzyme (New England Biolabs, United States) followed by whole genome amplification (WGA) of *Plasmodium* DNA by very high fidelity Phi29 DNA polymerase proofreading enzyme using the V2 DNA Amplification Kit (GE Lifesciences, Australia). The main aim of this protocol is to deplete contaminating human DNA in malaria field isolates by selectively digesting highly methylated DNA (human) followed by WGA of the remaining high molecular weight DNA (predominantly parasite). This enriches *Plasmodium* DNA for further use (e.g. SNP genotyping or whole genome sequencing). The protocol uses a minimum starting volume of 6ul of DNA extracted from human blood samples and works well even with low-density samples.

Primary PCR was performed to amplify target loci using optimized PCR#1 conditions as described in above. Then, primary PCR amplicons of each sample from two multiplex reactions were combined and purified using a QIAquick PCR Purification Kit (Qiagen) as per the manufacturer’s protocol. The amount of DNA in the primary PCR was measured using the Qubit DsDNA High Sensitivity (HS) Assay Kit (Thermo Fisher Scientific, Scoresby, Victoria, Australia) and normalized by diluting over-represented amplicons in PCR grade water. The secondary PCR reaction (PCR#2) was performed using cleaned primary PCR products as a template. Illumina adapters and a six-nucleotide sequence specific to each individual sample (MID index) was added to the template. The optimized conditions for PCR#2 requires 2 μl of combined primary PCR product in a 20 μl reaction consisting of 0.3 mM each dNTP, 3 mM MgCl_2_, 1X buffer (B1), 1 μM forward index primer, 1 μM reverse index primer and 1.5 unit of Hot Start DNA polymerase (QIAGEN). The PCR conditions consisted of an initial denaturation of 3 min at 95 °C followed by 25 cycles of 15 s at 95 °C, 30 s at 60 °C, and 30 s at 72 °C, and a final 7 min extension at 72 °C.

Equimolar amounts of each amplicon pool from all samples were combined into a single tube and purified using AMPure XP magnetic beads (Beckman Coulter) for library preparation. Standard sequencing libraries were prepared following the manufacturer’s recommended protocol and sequenced using in an Illumina MiSeq platform to generate (2X300) paired end reads. The TruSeq Custom Amplicon Sequencing Kit (Illumina, Inc) was used to allow 96 or more samples with integrated barcodes to be pooled prior to sequencing on an Illumina MiSeq.

## Data analysis

### Bioinformatic analysis

The raw FASTQ files were demultiplexed by binning based on the MID index, the read quality was checked using *FastQC* (Version 0.8.0) (https://www.bioinformatics.babraham.ac.uk/projects/fastqc/) and combined *FastQC* output for all samples were visualized using *MultiQC* [58]. Low-quality reads (< Q30), adaptors, primers, and reads shorter or longer than expected size of amplicon were trimmed using *Trimmomatic* [59]. Only reads that passed stringent quality filters progressed for alignment and variant calling.

Unmapped BAM files were generated from quality filtered and trimmed FASTQ files using the *FastqtoSam* function (http://broadinstitute.github.io/picard/). The combined pipeline was then used to generate indexed, mapped BAM files. This pipeline consists of *SamToFastq, bwa*-*mem* and *MergeBamAlignment* to map reads, and generated a clean and indexed mapped BAM file. In brief, the sequenced reads were mapped to the *P. vivax* Salvador I strain reference genome *bwa mem* [50] (Additional file 2: Figure S3). Overall quality and genome coverage of mapped bam files were checked using *QualiMap* v.2.2.1 [60]. We set a minimum cutoff of 50-fold coverage and successful genotyping of loci in at least 60% of sequenced samples to avoid inclusion of PCR and sequencing errors. After removing unsuccessfully amplified loci, the coverage and the frequencies of the reference and alternative alleles were determined using the *samtools mpileup* function [61] for each sample and SNP. A VCF file from the *samtools mpileup* analysis output was further filtered using vcftools to remove sites containing insertions and deletions [62]. Finally, all selected SNPs were further confirmed by visually inspecting the individual mapped reads using IGV software [63].

### Population genetic analyses

Alternative allele frequency (AAF) was computed as the proportion of genotyped samples whose genotype was not the reference allele for target loci. MAF was computed as the proportion of genotyped samples carrying the genotype that was least common (i.e. MAF = AAF if AAF < 0.5; MAF = (1-AAF) if not). To estimate the actual number of clones per sample the VCF file containing SNP data was converted to *The Real McCOIL* categorical method format: heterozygous call (0.5), homozygous minor allele (0), homozygous major allele (1) and no call (−1) and used as an input file for analysis of multiplicity of infection (MOI) using *The Real McCOIL* R package [63]. Input files for genetic analysis were created using the and PGDSpider (version 2.0.0.3) [65]. Genetic diversity was calculated as SNPπ using *DnaSP* Version 5.0 [64] for SNP data, and expected heterozygosity (*H*_*e*_) and allelic richness, using the *FSTAT* software, version 2.9.4 for microsatellite data [65]), were calculated. Genetic differentiation *(F*_**ST**_) was determined using *DnaSP* Version 5.0 [66] for SNP data and *FSTAT* 2.9.4 [67] for microsatellites. The Mantel Test was performed to measure associations between genetic distance and spatial geographical distance between catchments, using the R “Vegan” package [68]. Phylogenetic analysis was done based on the distance metric 1-*P*_S_ using the “Ape” R package and the ‘dist.gene’ function for SNP and microsatellite data and visualized using the *FigTree* software, version 1.4.3.

The Bayesian clustering software, *STRUCTURE* version 2.3.4 [69] was used to determine the number of discrete genetic clusters (K) and whether haplotypes cluster according to geographical origin. STRUCTURE runs were performed with a burn-in period of 100,000 followed by 100,000 Monte Carlo steps. The simulations were replicated 20 times with different seeds for *K* values ranging from 1 to 20. The optimal *K* value was calculated based on Evanno’s method of ΔK statistics. The *CLUMPAK* web-based server was used for summation and graphical representation of the STRUCTURE results. The assumptions underlying the population genetics model in *STRUCTURE* software may limit its use to detect malaria parasite population structure with declining transmission. Unlike natural populations, malaria parasites undergo inbreeding, clonal propagation, and there will be an absence of panmictic conditions when transmission declines. Therefore, to further explore parasite clustering the discriminant analysis of principal components (DAPC) was performed using the R package “Adegenet” [70]. DAPC is robust to Hardy–Weinberg disequilibrium or linkage disequilibrium [71].

### Statistical analysis

The Mann–Whitney U test or a one-way analysis of variance were used to measure differences among two groups or more than two groups, respectively. To assess the concordance between genotype allele sharing by SNPs and microsatellite markers we performed correlation analysis using *Kendall’s Tau*. Statistical analyses were performed using GraphPad Prism Software version 7.0 and a *p* value of ≤ 0.05 was considered statistically significant.

## Results

### Identification of SNP candidates

From a total of 40 *P. vivax* isolates from PNG, 23 were sequenced at the Broad Institute (BI) in Boston, MA, USA [14] and the remaining 17 were sequenced at the Wellcome Trust Sanger Institute (WTSI), Cambridge UK as part of the MalariaGEN *Plasmodium vivax* Community Project [51]. To include the highest quality samples for SNP selection, 16 isolates were excluded due to low quality and poor coverage (less than 90% of their bases covered up to 5x) or to remove the lowest quality genome of any duplicated samples (4 isolates). Additionally, four samples derived from sequencing pooled isolates were excluded from analysis since pooling could affect variant calling. The remaining 20 *P. vivax* genomes, originating from three hyper-endemic provinces of PNG (Madang = 17, East Sepik = 2, Milne Bay = 1, Fig. 1, Additional file 1: Table S1), were used to select informative SNPs. From a total of 405,825 variants present on 14 nuclear chromosomes, 144,517 were included after excluding all indel calls and ‘blacklisted’ highly polymorphic regions including the telomeres. Finally, after additional ‘hard filtering’ (see “Methods”), 220 SNPs with MAF greater than 10% and relatively uniformly spaced across *P. vivax* genome were selected for assay development (Additional file 2: Figure S1).

### Assay development

Six *P. vivax* field isolates were used to develop and optimize the new SNP genotyping assays. To evaluate the amplification of each target locus in the multiplex PCR, single-plex PCR was performed using primary multiplexed PCR products as the template. Of the six samples, three contained single clones (multiplicity of infection, MOI = 1) and the remaining three samples had two clones (MOI = 2) based on *Pvmsp1F3* and *Pvms16* genotyping [48]. Polyclonal infections were included to assess amplification bias of SNP alleles in complex infections due to multiple amplification steps. Of the 220 primer pairs tested, 178 produced a single clear band of the expected size. These 178 SNP loci were then used to develop a multiplex PCR, for further genotyping of *P. vivax* isolates from PNG (Additional file 3: Table S2). Only two SNPs from the previously developed barcode [33] met the inclusion criteria (Additional file 3: Table S2). To assess amplification bias that may occur due to preliminary amplification steps such as whole genome amplification (WGA), amplicon deep sequencing was performed for six samples with, and without, WGA for a test set of 20 SNP markers (2 × 10-plex PCRs). A total of 17 of 20 (85%) SNPs were successfully genotyped in these samples. There were no significant amplification differences before and after WGA (*p *= 0.13 for MOI = 1 samples and *p *= 0.92 for MOI = 2 (Mann–Whitney U test)) (Additional file 2: Figure S4a). There were also no observed discrepancies in the genotype calls between the WGA and non-WGA samples. There was an average read depth of 133 (range = 50–567) for test run samples (Additional file 2: Figure S4b). This read depth of approximately 133X is suitable to call variants however deeper sequencing is required to do downstream population genetic analysis with high confidence.

### Data summary and validation of the barcode

A total of 94 low complexity (MOI $\le$ 2) *P. vivax* samples from a cross-sectional survey conducted in four catchment areas of PNG (Madang Province: Mugil, Malala and Utu; East Sepik Province: Ilaita area) (Fig. 1) were then genotyped for all 178 SNPs using the parallel targeted amplicon sequencing assay. These samples had already been genotyped with ten microsatellite markers in a previous study [49]. A total of 28,934,460 reads were generated from the MiSeq Illumina run with a variable sequencing coverage across samples per locus (median = 563, range 56 –7586). Of the 178 SNPs, five were not amplified at all (no reads detected) and 34 had high missingness (no reads for > 40% of samples). Of the 94 genotyped samples, 83 were successfully genotyped for the remaining 146 SNP markers (Additional file 2: Figure S4c) indicating the genotyping success rate amongst samples was 88.2% (83/94) with an 82.1% (146/178) marker positivity rate. There were no identical genotypes, suggesting that the barcode is a unique identifier for *P. vivax* isolates from PNG. The SNPs generally had moderate minor allele frequencies (MAF) with 98% of SNP loci showing greater than 10% MAF (Additional file 2: Figure S5a). There were no private SNPs (unique to any one population). Published genotyping data for MOI [72] and microsatellite data for these *P. vivax* isolates [49] was then used to compare the population genetic metrics with the new SNP barcode data.

### The SNP barcode detects more multiple clone infections than classical genotyping for MOI

Despite previous ‘classical’ genotyping for MOI using *msp1F3* and *ms16* indicating the majority of samples were single clone infections (i.e. one allele at both markers [48]), all 83 samples showed at least one heterozygous call (two alleles found amongst the reads at a particular SNP locus), which is evidence of polyclonality (genotyping error is filtered out by the variant calling algorithm). *The Real McCOIL* analysis indicated that out of the 83 successfully genotyped *P. vivax* samples, 69 samples (83.2%) have at least two clones and 24 samples (16.8%) were confirmed monoclonal infections. For the population genetic analyses, the dominant allele was used to reconstruct dominant haplotypes (Additional file 4: Table S3).

### The SNP barcode captures variable genetic diversity amongst parasite populations

**Fig. 2:**
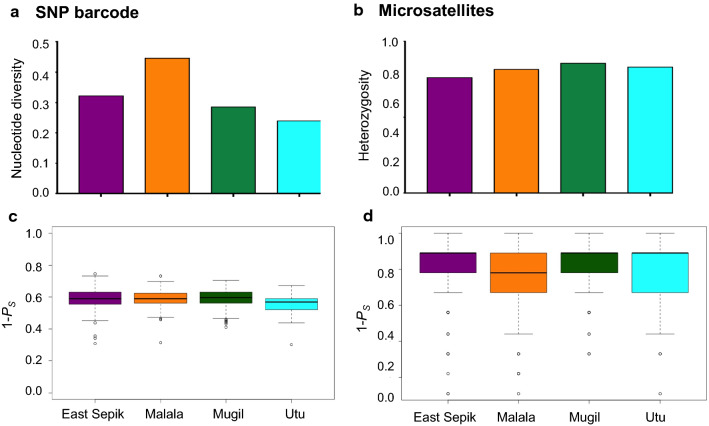
Genetic diversity of *P. vivax* populations from the north coast of Papua New Guinea. Genetic diversity was measured for four catchment areas on the north coast of Papua New Guinea using **a** SNP nucleotide diversity (π), which was measured by calculating the average number of pairwise differences at assayed SNPs between all members of sample using *DnaS*P Version 5.0 [65]; **b** Microsatellite Expected Heterozygosity (*He *= [n/(n-1)] [(1- Σp_i_^2^)] where n is the number of isolates sampled and p_i_ is the allele frequency at the ith loci) using as *FSTAT* software version 2.9.4 [67]; **c** SNP barcode diversity and **d** microsatellite haplotype diversity. For **c** and **d**, box plots show the results from another genetic diversity metric, 1-mean pairwise allele sharing. The variation in the box and median distribution indicates variability in genotype relatedness amongst pairs of genotypes. The analysis was done using genetic distance matrix for 1-*P*_S_ generated by the ‘dist. gene’ command in “Ape” R package

Variable levels of within-population genetic diversity were observed in the four parasite populations with an average nucleotide diversity (π) of 0.33 per SNP site (range = 0.24–0.45) (Fig. 2a). Nucleotide diversity was lowest in the inland Utu population and highest in the coastal Malala parasite population. The genetic diversity (Heterozygosity, *H*_*e*_) of the same parasite populations using the microsatellite panel showed uniformly high genetic diversity among populations (mean *H*_*e*_ = 0.82, range = 0.78–0.85) (Fig. 2b). Note that the different diversity measures necessary for these different markers may also impact this result. Therefore, we used the alternative metric 1-*P*_*S*_ (1-pairwise allele sharing) (Fig. 2c, d) to measure genetic diversity within each parasite population for both markers. Genetic diversity by both marker panels was significantly different among the four parasite populations (*p value *< 0.001, Kruskal–Wallis test). In general, microsatellite genotypes had higher genetic diversity compared to SNP genotypes. However, microsatellites show a wider range of values and more closely related genotype pairs (outliers 1-*Ps* < 0.4) (Fig. 2d).

### The SNP barcode detects parasite population divergence that is associated with geographic distance

Bayesian cluster analysis of SNP genotypes using *STRUCTURE* software [66] identified that three genetic clusters (K = 3) provided the best fit for the SNP data and two genetic clusters (K = 2) for microsatellites (Additional file 2: Figure S6).

**Fig. 3:**
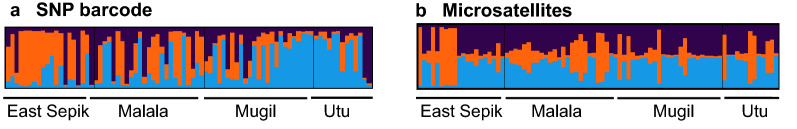
Bayesian cluster analysis of *P. vivax* genotypes from the north coast of Papua New Guinea. Cluster analysis was done using **a** SNP barcodes or **b** microsatellite haplotypes for 86 *P. vivax* isolates from four geographic regions of Papua New Guinea using STRUCTURE software version 2.3.4 [68]. STRUCTURE bar plots representing Individual ancestry coefficients are shown for K = 3, each vertical bar represents an individual haplotype and the membership coefficient (Q) within each of the genetic populations, as defined by the different colours

**Fig. 4:**
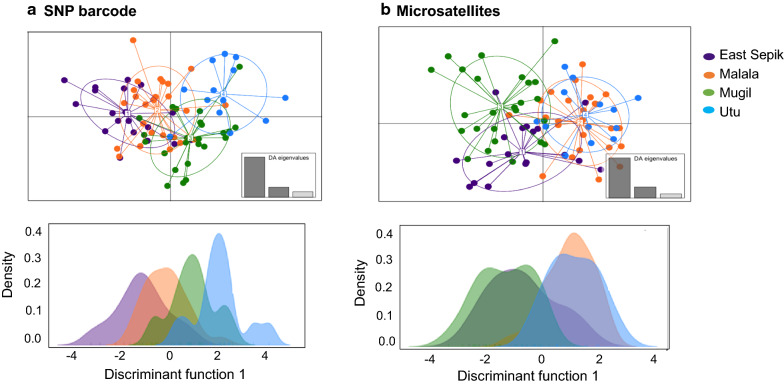
Discriminant analysis of principal component (DAPC) of *P. vivax* isolates from the north coast of Papua New Guinea. DAPC was used to identify clustering amongst isolates from the four catchment areas for **a** SNP barcodes and **b** microsatellite haplotypes. On the top of the figure scatterplots of DAPC (Bottom) are shown. Clusters are defined by ellipses and indicate the variance within the clusters whereas dots indicate the positions of individual parasite genotypes within the cluster. Eigenvalues represent the amount of genetic variation captured by the discriminant factors plotted as the x- and y-axis. On the bottom, individual density plots are shown for the first discriminant function. The data was analysed using DAPC function in “Adegenet” R package [69]

Less population structure and genotype clustering according to their geographic origin was observed by microsatellite markers compared to SNPs (Fig. 3). Discriminant Analysis of Principal Components (DAPC) detected higher levels of genotype assignment to different geographic origins and higher differentiation between distant compared to neighbouring populations for SNPs (Fig. 4a, top) than microsatellite markers (Fig. 4b, top). Microsatellites revealed limited differentiation of distant parasite populations such as East Sepik and Utu (Fig. 4b, top).

To further explore the patterns of gene flow in different geographic areas we measured genetic differentiation (*F*_ST_) and observed very low to moderate genetic differentiation (*F*_ST_ = 0.02–0.12) between parasite populations using either marker panel (Table 1). However, values were higher for SNPs and there was greater differentiation of distant populations e.g. East Sepik vs Utu for the SNP marker data.

**Table 1:** Pairwise population differentiation among *P. vivax* populations in four different geographic clusters in North Coast of Papua New Guinea

Population	East Sepik	Malala	Mugil	Utu
East Sepik	–	0.025	0.033	0.12
Malala	0.09	–	0.06667	0.045
Mugil	0.086	0.021	–	0.0833
Utu	0.121	0.04033	0.033	–

Lower left = SNP F_*ST*_, Upper right = Microsatellite F_*ST*_

**Fig. 5:**
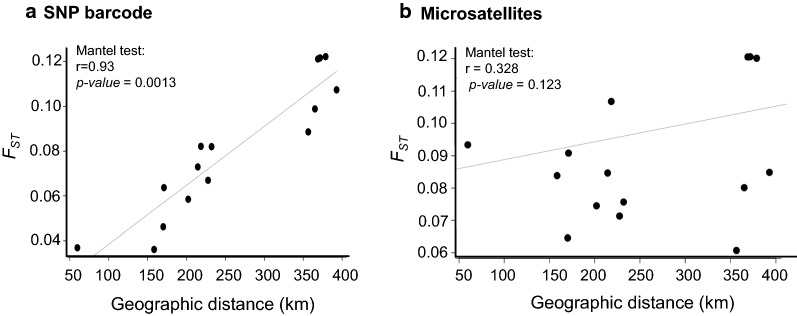
Association between geographic and genetic distance between *P. vivax* populations of Papua New Guinea. Genetic differentiation between population pairs was measuring using *F*_ST_ for **a** SNP barcodes and **b** microsatellites and measured in association with geographic distance based on geographic co-ordinates of villages. A Mantel test was used to measure the association using R “Vegan” package [67]

For SNP barcodes, the DAPC individual density plot also supports the *F*_ST_ result, where distant parasite populations were more distinctly clustered (Fig. 4a, bottom) than the nearby populations. However, the microsatellite marker data showed an unusual clustering of distant parasite populations together (East Sepik and Utu) (Fig. 4b, bottom). To assess whether the geographic distance between geographic clusters affects gene flow, a Mantel correlation test was conducted. The analysis showed a significant association between genetic distance and geographic distance in km for SNP markers (Fig. 5a), but not for microsatellite markers (Fig. 5b).

### No association between microsatellite and SNP haplotype relatedness

**Fig. 6:**
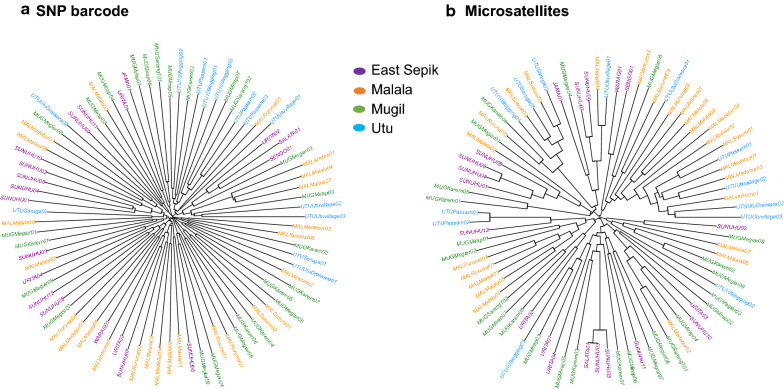
Phylogenetic relationships among individual *P. vivax* isolates from the north coast of Papua New Guinea. Neighbour joining trees are shown for **a** SNP barcodes and **b** microsatellite haplotypes. Branches are coloured according to the four catchment areas. Labels indicate a unique sample ID and village of origin. Distance was measured using the genetic distance matrix for 1-*P*_*S*_ calculated by “Ape” R package, ‘dist. gene’ command

To further explore clustering patterns and investigate the relatedness of individual SNP haplotypes, phylogenetic analysis was conducted using Neighbour Joining trees. This identified clusters of closely related isolates from the same province and village with moderate population structure and geographic clustering of genotypes (Fig. 6). More clustering of genotypes was found in the East Sepik population compared to the three parasite populations from Madang (Malala, Mugil and Utu for the SNP markers) (Fig. 6a). Overall, phylogenetic analysis supported the *STRUCTURE* and *DAPC* results, with higher parasite clustering between East Sepik versus Madang by SNP barcode compared to microsatellites.

Unless there is overall high relatedness among genotypes, it is difficult to identify population structure using phylogenetic analysis due to high recombination between distinct clones. To further infer relatedness between parasites within and between populations, a simple pairwise allele-sharing (*P*_*S*_) measure was used. Relatedness analysis using the SNP markers (Additional file 2: Figure S6) showed that the majority of genotypes share alleles at 50–70% of markers suggesting parasites are unrelated [73]. Only a few genotypes showed high relatedness, with 70–90% of alleles shared (Additional file 2: Figure S7) within the population, but no identical genotypes were detected. The allele sharing analysis of the same *P. vivax* isolates using ten microsatellite markers was consistent with SNP data where the majority of microsatellite genotypes are unique and only a few genotypes shared a high proportion of alleles.

Concordance analysis of allele sharing between genotypes using the SNP barcode and microsatellite haplotypes showed no statistically significant association (r = 0.032, *p*-*value *= 0.56) (Additional file 2: Figure S8). Thus, high outcrossing in the PNG populations due to high transmission removes any association between these markers.

## Discussion

Genomic surveillance of malaria parasite populations is a useful tool to assess changing transmission patterns, identify imported cases and track the spread of infections [23, 74, 75]. Reliable, cheap and high-resolution genotyping assays are therefore needed to support malaria control programmes. SNP barcodes have been developed to study the complexity of infection [76, 77], parasite population structure and the origins of outbreaks [15, 33, 34, 78]. However, ascertainment bias can reduce the sensitivity of detecting distinct clones and population genetic analyses to detect and track discrete parasite populations. SNP barcodes need to be validated and/or tailored to specific geographic areas to reflect the SNP diversity in local parasite populations [23]. Here, the development of a SNP barcode comprising 178 locally-validated biallelic SNP loci is described. This barcode was tailored specifically to PNG, one of the world’s hotspots for *P. vivax* malaria infection. Genetic diversity and population structure amongst four distinct catchment areas on the north coast of PNG was compared for the SNP barcode and panel of ten polyallelic microsatellite markers that many groups have previously employed for population genetic analyses [24–26]. The results demonstrate the greater sensitivity of these large biallelic SNP barcodes for malaria genomic epidemiology and potential to provide useful data to guide malaria control strategies.

The SNP barcode detected a higher number of clones compared to two highly polymorphic microsatellite markers *ms16* and *msp1F3*, which have been used previously to measure multiplicity of infection [79, 80]. This indicates that the SNP barcode has higher resolution to identify multiple infections, most likely due to the much larger number of loci genotyped. However, there is an upper limit to clone detection due to the biallelic nature of the SNP loci. It also suggests that the complexity of *P. vivax* infections (based on a small number of loci) is currently underappreciated, and that the barcode will be more useful than small numbers of microsatellites in very low endemic settings to distinguish between very closely related parasites. Samples were pre-genotyped and selected for monoclonal infection, which limits the direct comparison of the ability of these two microsatellites and SNP barcode to identify polyclonal infections. Further evaluation of the barcode by genotyping a large set of randomly selected field samples is needed to fully assess its utility for estimating complexity of infection.

Population genetic analyses using the SNP barcode elucidated genetic diversity, relatedness, population structure and connectivity of circulating parasite populations at higher resolution relative to the larger panel of ten microsatellite markers. More variable genetic diversity among populations is captured by the SNP barcode than microsatellite markers. Previous work with microsatellite markers in eight locations of PNG revealed geographic population structure between the mainland, islands and highland areas [9]. However, microsatellites were unable to differentiate populations at a finer spatial scale between the mainland north coast provinces of East Sepik and Madang [46, 47, 49]. Indeed, microsatellite performance has not previously been compared to SNPs in terms of their ability to differentiate between *P. vivax* populations. The results suggest that large numbers of SNPs have higher resolution to detect differences in transmission dynamics between populations. Moreover, SNP barcodes detected substantial geographic population structure between the four catchment areas with clustering of haplotypes according to their geographic origin, whereas microsatellites did not achieve this. There was also a significant association between genetic distance and geographic distance for SNPs but not for microsatellite markers suggesting that SNPs can accurately pinpoint geographic origins of infections, whereas microsatellites cannot. This also implies that large numbers of SNP markers can capture population connectivity at fine spatial scales in high transmission areas. The ten microsatellite markers identify some population structure, but it does not fit the expected isolation by distance pattern expected of this contiguous endemic area. The findings are consistent with another study on the malaria vector *Anopheles darlingi* where SNP markers showed higher discrimination among genetic clusters with more than 4–35 fold higher *F*_*ST*_ estimates than microsatellite markers [81]. Other studies in different fish species have also shown that biallelic SNP markers have greater accuracy and finer population structure than microsatellite markers [82, 83]. The SNP barcode is more sensitive because markers are more densely covering the chromosomes than the microsatellite panel with less than one marker per chromosome, and thus will more accurately detect relatedness among parasites through inherited segments of the genome, using Identity by Descent (IBD) approaches [84, 85]. Also, in high transmission areas, genetic differentiation (i.e. the difference between the diversity of the subpopulations compared to the metapopulation [86]) is typically estimated as being low when using polyallelic microsatellite markers because their diversity is at a maximum, making it difficult to identify low levels of population structure. Other measures such as *Jost’s D* have been used to overcome these limitations of microsatellites [47]. While our results suggest that the currently used ten microsatellite panel may have lower resolution to identify distinct genotypes and correctly identify related parasites, larger microsatellite panels will undoubtedly be more sensitive and may also deconvolute complex mixtures of clones within an infection. Selection of informative markers is important to track gene flow and quantify parasite connectivity using IBD measures [87]. SNP barcoding using an adequate number and density of SNPs will be important for the characterization of these population genetic signals, and to identify patterns of parasite migration [23, 32, 87]. The generation of additional WGS data for the surveyed populations would help to verify the performance of different marker panels.

Related isolates have a higher probability of identical alleles at a given locus than unrelated genotypes [73, 74]. In this study, a significant difference in pairwise allele sharing was not detected either within and between populations using SNP or microsatellite markers. The SNP barcode identified a narrow range of allele sharing with > 90% genotypes with alleles shared amongst 50–90% of the markers. This finding is consistent with the previous study by Nkhoma et al. [73] where a SNP barcode comprising 96 SNPs detected high allele sharing in diverse and unrelated parasites (*P*_S_ of up to 0.74 observed in two unrelated parasites (0% IBD)). Thus, only genotypes with *P*_S_ values greater than 0.8 (distributed at tail end of the histogram) for either SNP or microsatellite markers are truly related. This finding indicates that simple pairwise allele sharing (IBS) values do not accurately represent the actual percentage of the parasite genome that is IBD or that this measure is not as sensitive to measure the actual genotype relatedness. IBD values were not compared between the different markers in this study since the ten microsatellite markers will have limited sensitivity to estimate the proportion of the genome that is IBD. Studies using large SNP barcodes (> 100 loci) such as that described here, are recommended in order to apply IBD measures to calculate parasite relatedness and between population connectivity [87].

A minor allele frequency criteria was applied to select informative SNPs (> 0.1 MAF) to capture diverse parasites in hyperendemic regions of PNG. It is recommended to use these validated (n = 146) bi-allelic SNPs for future genotyping of *P. vivax* parasites if informative for the studied parasite population. A similar approach has been used to select a ‘universal’ 42 SNP barcode from hundreds of thousands of SNPs from genomic sequences of globally diverse parasite isolates [33]. A recent study focused on *Plasmodium falciparum* revealed that barcodes of 93 or even just 24 SNP markers are adequate in a low transmission area to capture parasite connectivity, allow stratification of closely related parasite populations and identify source and sink populations—with a high sample size required for a small number of markers and vice versa [85]. Before applying the developed markers to genotype parasite isolates from a given population, it is recommended to validate markers by evaluating allele frequencies within a subset of samples. Moreover, SNP barcodes need to be continually evaluated and validated against local WGS data to ensure they provide similar insights into parasite population genetics.

In conclusion, the locally-validated SNP barcoding assay showed higher resolution to measure variations in *P. vivax* diversity and population structure at local (sub-provincial) scale compared to the currently used panel of ten microsatellite markers. As countries approach malaria elimination, SNP barcoding will help to identify transmission zones and their dynamics and routes of parasite migration, and hence how to contain infections and to monitor whether control efforts are having an impact. The findings from this approach in combination with epidemiological data are essential to policy makers. The developed amplicon sequencing assay requires only a small amount of starting DNA (2 mL) and can be done relatively easily using available Next Generation Sequencing technology platforms at low cost (less than $18USD per isolate). This technology allows the “plug and play” incorporation of other markers such as SNPs informative for a given country/region or those associated with drug resistance that could help to concurrently genotype circulating parasites and resistance genes to give timely information for malaria control strategies. Overall, the findings suggest that SNPs may be better suited than the currently used microsatellite markers due to their higher resolution. SNP barcodes would also be more suitable in a control program setting, given the availability of cost-effective and robust high-throughput sequencing and the relative lack of technical issues.

## Supplementary information

**Additional file 1: Table S1.** Details of 40 *P. vivax* isolates from PNG sequenced and used for SNP candidate selection.

**Additional file 2: Figure S1.** Overview of steps used to select informative SNPs from *P. vivax* whole genome sequence data. **Figure S2.** Overview of amplicon sequencing approach for multiple samples (96) to amplify all target SNP loci (178) in single sequencing run. Genomic loci were amplified using standard PCR (PCR#1) using locus specific primer with universal overhang adaptors (**a**). Using purified primary PCR product as a template an additional PCR, index PCR (PCR#2) (**b**) was performed to attach a multiplex identifier (MID) tag which is unique to each sample and attaches to universal overhang sequence. Unique sequence of MID for each sample enables pooling of secondary PCR products (96 samples in this protocol) for library preparation (**c**). Then high throughput “multiplexed” sequencing of the combined amplicons from all samples (96) in a single MiSeq run to produce require millions of paired-end reads (2x300bp) (**d**). **e** Data was analysed using standard bioinformatics tools and mapped reads were visualized using Integrative Genomics Viewer. The graph shows a large number of reads covering the target SNP locus. This result shows that all reads possess the alternative allele (green) at the SNP site. This high frequency of SNP calls amongst reads gives high confidence to differentiate true SNPs (indicated as SNPS at target locus) from sequencing artefacts (rare SNPs shown left and right side of target locus). **Figure S3.** Overview of bioinformatic data analysis pipeline. Data processing sequence read consisting of quality checking of raw sequence reads, primers and adaptor trimming, mapping reads to reference sequence, SNP calling and filtering, and population genetic and statistical analyses. **Figure S4.** Quality control of the *Plasmodium vivax* barcoding assay. **a** Assessment of amplification bias and SNP polymorphism among genotyped samples. Comparison of number of successfully amplified loci before and after rWGA of samples. There was no statistically significant difference in number of loci successfully genotyped before and after WGA, and between monoclonal and polyclonal samples. **b** Read coverage for all 20 SNP markers per sequenced sample. The graph shows that read count is different for different samples with high read count in samples with MOI = 2. Read count variation between amplified WGA) and unamplified samples (S) were not significant. **c** PCR and SNP genotyping success rate. From a total of 220 SNPs, 42 were negative and were not amplified in the primary multiplex PCR, and 32 failed during genotyping (either no reads detected or did not meet the quality filtering threshold). The remaining 146 were used as “SNP barcode” for downstream population genetic analysis (Additional file 3: Table S2). **Figure S5.** Distribution of the Minor allele frequency (MAF) of SNP barcodes in *P. vivax* parasite population in north coast PNG. **Figure S6.** Optimal number of clusters for each marker based Evanno’s method [42]. For SNP markers the method identified three genetic clusters (K = 3) and two genetic clusters (K = 2) for Microsatellites. However, for Microsatellites K = 2 is a common artifact of the hierarchical clustering algorithm when two very distinct populations are present, so higher K must be observed to identify possible sub-population structure. **Figure S7.** Pairwise allele sharing of *P. vivax* genotypes within and between population. **a** SNP genotype frequency distribution of pairwise allele-sharing (*P*_S_). **b** Microsatellite genotype frequency distribution of pairwise allele-sharing (*P*_S_). Black bars indicate within population and grey bars indicate between populations. There was no significant difference in allele sharing within and between parasite populations either SNP barcode or microsatellite markers in north coast of PNG. **Figure S8**. Kendall’s Tau concordance analysis of genotype allele sharing by SNP and Microsatellite. Each boxplot indicates the proportion of shared alleles between genotypes by SNP. The analysis revealed no significant correlation between the SNP and microsatellite pairwise allele sharing values.

**Additional file 3: Table S2.** Details of 178 SNPs, SNPs included in final panel (n = 146), primers sequences and their multiplex setting.

**Additional file 4: Table S3.** The 83 *P. vivax* haplotypes used for the final data analysis.

## Data Availability

Raw genotyping datasets used and/or analysed during the current study are available from the corresponding author on reasonable request. All final genotypes analysed during this study are included in its Additional files.

## References

[1] WHO. World malaria report 2019. Geneva: World Health Organization; 2019.

[2] Karyana M, Burdarm L, Yeung S, Kenangalem E, Wariker N, Maristela R (2008). Malaria morbidity in Papua Indonesia, an area with multidrug resistant *Plasmodium vivax* and *Plasmodium falciparum*. Malar J.

[3] Quispe AM, Pozo E, Guerrero E, Durand S, Baldeviano GC, Edgel KA (2014). *Plasmodium vivax* hospitalizations in a monoendemic malaria region: severe vivax malaria?. Am J Trop Med Hyg.

[4] Tjitra E, Anstey NM, Sugiarto P, Warikar N, Kenangalem E, Karyana M (2008). Multidrug-resistant *Plasmodium vivax* associated with severe and fatal malaria: a prospective study in Papua Indonesia. PLoS Med.

[5] Price RN, Seidlein L, Valecha N, Nosten F, Baird JK, White NJ (2014). Global extent of chloroquine-resistant *Plasmodium vivax*: a systematic review and meta-analysis. Lancet Infect Dis.

[6] Mueller I, Galinski MR, Baird JK, Carlton JM, Kochar DK, Alonso PL (2009). Key gaps in the knowledge of *Plasmodium vivax*, a neglected human malaria parasite. Lancet Infect Dis.

[7] Adams JH, Mueller I (2017). The biology of *Plasmodium vivax*. Cold Spring Harb Perspect Med.

[8] Wang D, Li S, Cheng Z, Xiao N, Cotter C, Hwang J (2015). Transmission risk from imported *Plasmodium vivax* malaria in the China-Myanmar border region. Emerg Infect Dis.

[9] Fola AA, Nate E, Abby Harrison GL, Barnadas C, Hetzel MW, Iga J (2018). Nationwide genetic surveillance of *Plasmodium vivax* in Papua New Guinea reveals heterogeneous transmission dynamics and routes of migration amongst subdivided populations. Infect Genet Evol.

[10] Ohrt C, Roberts KW, Sturrock HJW, Wegbreit J, Lee BY, Gosling RD (2015). Information systems to support surveillance for malaria elimination. Am J Trop Med Hyg.

[11] Barclay VC, Smith RA, Findeis JL (2012). Surveillance considerations for malaria elimination. Malar J.

[12] Rosanas-Urgell A, Mueller D, Betuela I, Barnadas C, Iga J, Zimmerman P (2010). Comparison of diagnostic methods for the detection and quantification of the four sympatric *Plasmodium* species in field samples from Papua New Guinea. Malar J.

[13] Arnott A, Barry AE, Reeder JC (2012). Understanding the population genetics of *Plasmodium vivax* is essential for malaria control and elimination. Malar J.

[14] Pearson RD, Amato R, Auburn S, Miotto O, Almagro-Garcia J, Amaratunga C (2016). Genomic analysis of local variation and recent evolution in *Plasmodium vivax*. Nat Genet.

[15] Obaldia N, Baro NK, Calzada JE, Santamaria AM, Daniels R, Wong W (2015). Clonal outbreak of *Plasmodium falciparum* infection in eastern Panama. J Infect Dis.

[16] Preston MD, Campino S, Assefa SA, Echeverry DF, Ocholla H, Amambua-Ngwa A (2014). A barcode of organellar genome polymorphisms identifies the geographic origin of *Plasmodium falciparum* strains. Nat Commun.

[17] Auburn S, Benavente ED, Miotto O, Pearson RD, Amato R, Grigg MJ (2018). Genomic analysis of a pre-elimination Malaysian *Plasmodium vivax* population reveals selective pressures and changing transmission dynamics. Nat Commun.

[18] Volkman SK, Neafsey DE, Schaffner SF, Park DJ, Wirth DF (2012). Harnessing genomics and genome biology to understand malaria biology. Nat Rev Genet.

[19] Neafsey DE, Volkman SK (2017). Malaria genomics in the era of eradication. Cold Spring Harb Perspect Med.

[20] Escalante AA, Ferreira MU, Vinetz JM, Volkman SK, Cui L, Gamboa D (2015). Malaria molecular epidemiology: lessons from the International Centers of Excellence for Malaria Research Network. Am J Trop Med Hyg.

[21] Omedo I, Mogeni P, Rockett K, Kamau A, Hubbart C, Jeffreys A (2017). Geographic-genetic analysis of *Plasmodium falciparum* parasite populations from surveys of primary school children in Western Kenya. Wellcome Open Res.

[22] Bousema T, Drakeley C, Gesase S, Hashim R, Magesa S, Mosha F (2010). Identification of hot spots of malaria transmission for targeted malaria control. J Infect Dis.

[23] Daniels RF, Rice BL, Daniels NM, Volkman SK, Hartl DL (2015). The utility of genomic data for *Plasmodium vivax* population surveillance. Pathog Glob Health.

[24] Karunaweera ND, Ferreira MU, Hartl DL, Wirth DF (2006). Fourteen polymorphic microsatellite DNA markers for the human malaria parasite *Plasmodium vivax*. Mol Ecol Notes.

[25] de Souza AM, de Araujo FC, Fontes CJ, Carvalho LH, de Brito CF, de Sousa TN (2015). Multiple-clone infections of *Plasmodium vivax*: definition of a panel of markers for molecular epidemiology. Malar J.

[26] Gunawardena S, Karunaweera ND, Ferreira MU, Phone-Kyaw M, Pollack RJ, Alifrangis M (2010). Geographic structure of *Plasmodium vivax*: microsatellite analysis of parasite populations from Sri Lanka, Myanmar, and Ethiopia. Am J Trop Med Hyg.

[27] Anderson TJ, Su XZ, Bockarie M, Lagog M, Day KP (1999). Twelve microsatellite markers for characterization of *Plasmodium falciparum* from finger-prick blood samples. Parasitology.

[28] Sutton PL (2013). A call to arms: on refining *Plasmodium vivax* microsatellite marker panels for comparing global diversity. Malar J.

[29] Madesis P, Ganopoulos I, Tsaftaris A (2013). Microsatellites: evolution and contribution. Methods Mol Biol.

[30] Havryliuk T, Orjuela-Sánchez P, Ferreira MU (2008). *Plasmodium vivax*: microsatellite analysis of multiple-clone infections. Exp Parasitol.

[31] McTavish EJ, Hillis DM (2015). How do SNP ascertainment schemes and population demographics affect inferences about population history?. BMC Genomics..

[32] Ferreira MU, Rodrigues PT (2014). Tracking malaria parasites in the eradication era. Trends Parasitol.

[33] Baniecki ML, Faust AL, Schaffner SF, Park DJ, Galinsky K, Daniels RF (2015). Development of a single nucleotide polymorphism barcode to genotype *Plasmodium vivax* infections. PLoS Negl Trop Dis.

[34] Daniels R, Volkman SK, Milner DA, Mahesh N, Neafsey DE, Park DJ (2008). A general SNP-based molecular barcode for *Plasmodium falciparum* identification and tracking. Malar J.

[35] Ball AD, Stapley J, Dawson DA, Birkhead TR, Burke T, Slate J (2010). A comparison of SNPs and microsatellites as linkage mapping markers: lessons from the zebra finch (*Taeniopygia guttata*). BMC Genomics.

[36] Rogers AR, Jorde LB (1996). Ascertainment bias in estimates of average heterozygosity. Am J Hum Genet.

[37] Nielsen R (2004). Population genetic analysis of ascertained SNP data. Hum Genomics.

[38] Lachance J, Tishkoff SA (2013). SNP ascertainment bias in population genetic analyses: why it is important, and how to correct it. BioEssays.

[39] Sunyaev SR, Lathe WC, Ramensky VE (2000). SNP frequencies in human genes: an excess of rare alleles and differing modes of selection. Trends Genet.

[40] Howes RE, Battle KE, Mendis KN, Smith DL, Cibulskis RE, Baird JK (2016). Global epidemiology of *Plasmodium vivax*. Am J Trop Med Hyg.

[41] Hetzel MW, Morris H, Tarongka N, Barnadas C, Pulford J, Makita L (2015). Prevalence of malaria across Papua New Guinea after initial roll-out of insecticide-treated mosquito nets. Trop Med Int Health.

[42] Koepfli C, Rodrigues PT, Antao T, Orjuela-Sanchez P, Van den Eede P, Gamboa D (2015). *Plasmodium vivax* diversity and population structure across four continents. PLoS Negl Trop Dis.

[43] Delgado-Ratto C, Gamboa D, Soto-Calle VE, Van den Eede P, Torres E, Sanchez-Martinez L (2016). Population genetics of *Plasmodium vivax* in the Peruvian Amazon. PLoS Negl Trop Dis.

[44] Getachew S, To S, Trimarsanto H, Thriemer K, Clark TG, Petros B (2015). Variation in complexity of infection and transmission stability between neighbouring populations of *Plasmodium vivax* in Southern Ethiopia. PLoS ONE.

[45] Waltmann A, Koepfli C, Tessier N, Karl S, Fola A, Darcy AW (2018). Increasingly inbred and fragmented populations of *Plasmodium vivax* associated with the eastward decline in malaria transmission across the Southwest Pacific. PLoS Negl Trop Dis.

[46] Koepfli C, Timinao L, Antao T, Barry AE, Siba P, Mueller I (2013). A large *Plasmodium vivax* reservoir and little population structure in the South Pacific. PLoS ONE.

[47] Jennison C, Arnott A, Tessier N, Tavul L, Koepfli C, Felger I (2015). *Plasmodium viva*x populations are more genetically diverse and less structured than sympatric *Plasmodium falciparum* populations. PLoS Negl Trop Dis.

[48] Koepfli C, Ome-Kaius M, Jally S, Malau E, Maripal S, Ginny J (2017). Sustained malaria control over an eight-year period in Papua New Guinea: the challenge of low-density asymptomatic infections. J Infect Dis.

[49] Kattenberg JH, Razook Z, Keo R, Koepfli C, Jennison C, Lautu-Ninda D, et al. Monitoring of *Plasmodium falciparum* and *Plasmodium vivax* using microsatellite markers indicates limited changes in population structure after substantial transmission decline in Papua New Guinea. Mol Ecol. 2020; 10.1111/mec.15654 ahead of print. 10.1111/mec.15654 PMC10008436 32985031

[50] McKenna A, Hanna M, Banks E, Sivachenko A, Cibulskis K, Kernytsky A (2010). The Genome Analysis Toolkit: a mapReduce framework for analyzing next-generation DNA sequencing data. Genome Res.

[51] Hupalo DN, Luo Z, Melnikov A, Sutton PL, Rogov P, Escalante A (2016). Population genomics studies identify signatures of global dispersal and drug resistance in *Plasmodium vivax*. Nat Genet.

[52] Carlton JM, Adams JH, Silva JC, Bidwell SL, Lorenzi H, Caler E (2008). Comparative genomics of the neglected human malaria parasite *Plasmodium vivax*. Nature.

[53] Li H, Durbin R (2009). Fast and accurate short read alignment with Burrows-Wheeler transform. Bioinformatics.

[54] Lu J, Johnston A, Berichon P, Ru K-l, Korbie D, Trau M. PrimerSuite: a hig-throughput web-Based primer design program for multiplex bisulfite PCR. Sci Rep. 2017;7:41328. 10.1038/srep41328 PMC5259761 28117430

[55] Henegariu O, Heerema NA, Dlouhy SR, Vance GH, Vogt PH (1997). Multiplex PCR: critical parameters and step-by-step protocol. Biotechniques.

[56] Holbrook JF, Stabley D, Sol-Church K (2005). Exploring whole genome amplification as a DNA recovery tool for molecular genetic studies. J Biomol Tech.

[57] Hosono S, Faruqi AF, Dean FB, Du Y, Sun Z, Wu X (2003). Unbiased whole-genome amplification directly from clinical samples. Genome Res.

[58] Ewels P, Magnusson M, Lundin S, Käller M (2016). MultiQC: summarize analysis results for multiple tools and samples in a single report. Bioinformatics.

[59] Bolger AM, Lohse M, Usadel B (2014). Trimmomatic: a flexible trimmer for Illumina sequence data. Bioinformatics.

[60] García-Alcalde F, Okonechnikov K, Carbonell J, Cruz LM, Götz S, Tarazona S (2012). Qualimap: evaluating next-generation sequencing alignment data. Bioinformatics.

[61] Li H, Handsaker B, Wysoker A, Fennell T, Ruan J, Homer N (2009). The sequence alignment/map format and SAMtools. Bioinformatics.

[62] Danecek P, Auton A, Abecasis G, Albers CA, Banks E, DePristo MA (2011). The variant call format and VCFtools. Bioinformatics.

[63] Robinson JT, Thorvaldsdóttir H, Winckler W, Guttman M, Lander ES, Getz G (2011). Integrative Genomics Viewer. Nat Biotechnol.

[64] Chang HH, Worby CJ, Yeka A, Nankabirwa J, Kamya MR, Staedke SG (2017). THE REAL McCOIL: a method for the concurrent estimation of the complexity of infection and SNP allele frequency for malaria parasites. PLoS Comput Biol.

[65] Lischer HEL, Excoffier L (2012). PGDSpider: an automated data conversion tool for connecting population genetics and genomics programs. Bioinformatics.

[66] Librado P, Rozas J (2009). DnaSP v5: a software for comprehensive analysis of DNA polymorphism data. Bioinformatics.

[67] Goudet J. FSTAT (version 2.9.4), a program to estimate and test population genetics parameters. 2003. http://www.t-de-meeus.fr/Programs/Fstat294.zip. Updated from Goudet [1995].

[68] Dixon P (2003). VEGAN, A Package of R Functions for Community Ecology. J Veg Sci.

[69] Pritchard JK, Stephens M, Donnelly P (2000). Inference of population structure using multilocus genotype data. Genetics.

[70] Jombart T (2008). Adegenet: a R package for the multivariate analysis of genetic markers. Bioinformatics.

[71] Jombart T, Devillard S, Balloux F (2010). Discriminant analysis of principal components: a new method for the analysis of genetically structured populations. BMC Genet.

[72] Katterberg JH, Gumal DL, Ome-Kaius M, Kiniboro B, Philip M, Jally S (2020). The epidemiology of *Plasmodium falciparum* and *Plasmodium vivax* in East Sepik Province Papua New Guinea, pre- and post-implementation of national malaria control efforts. Malar J.

[73] Nkhoma SC, Nair S, Cheeseman IH, Rohr-Allegrini C, Singlam S, Nosten F (2012). Close kinship within multiple-genotype malaria parasite infections. Proc R Soc Bio Sci.

[74] Omedo I, Mogeni P, Bousema T, Rockett K, Amambua-Ngwa A, Oyier I (2017). Micro-epidemiological structuring of *Plasmodium falciparum* parasite populations in regions with varying transmission intensities in Africa. Wellcome Open Res.

[75] Nkhoma SC, Nair S, Al-Saai S, Ashley E, McGready R, Phyo AP (2013). Population genetic correlates of declining transmission in a human pathogen. Mol Ecol.

[76] Daniels R, Chang HH, Séne PD, Park DC, Neafsey DE, Schaffner SF (2013). Genetic surveillance detects both clonal and epidemic transmission of malaria following enhanced intervention in Senegal. PLoS ONE.

[77] Daniels RF, Schaffner SF, Wenger EA, Proctor JL, Chang HH, Wong W (2015). Modeling malaria genomics reveals transmission decline and rebound in Senegal. Proc Natl Acad Sci USA.

[78] Volkman SK, Ndiaye D, Diakite M, Koita O, Nwakanma D, Daniels R (2012). Application of genomics to field investigations of malaria by the International Centers for Excellence in Malaria Research. Acta Trop.

[79] Fola AA, Abby Harrison GL, Hazairin MH, Barnadas C, Hetzel MW, Iga J (2017). Higher complexity of infection and genetic diversity of *Plasmodium vivax* than *Plasmodium falciparum* across all malaria transmission zones of Papua New Guinea. Am J Trop Med Hyg.

[80] Waltmann A, Darcy AW, Harris I, Koepfli C, Lodo J, Vahi V (2015). High Rates of Asymptomatic, sub-microscopic *Plasmodium vivax* infection and disappearing *Plasmodium falciparum* malaria in an area of low transmission in Solomon Islands. PLoS Negl Trop Dis.

[81] Campos M, Conn JE, Alonso DP, Vinetz JM, Emerson KJ, Ribolla PE (2017). Microgeographical structure in the major Neotropical malaria vector *Anopheles darlingi* using microsatellites and SNP markers. Parasit Vectors.

[82] Ryynanen HJ, Tonteri A, Vasemagi A, Primmer CR (2007). A comparison of biallelic markers and microsatellites for the estimation of population and conservation genetic parameters in Atlantic salmon (*Salmo salar*). J Hered.

[83] Jeffries DL, Copp GH, Lawson Handley L, Olsen KH, Sayer CD, Hanfling B (2016). Comparing RADseq and microsatellites to infer complex phylogeographic patterns, an empirical perspective in the Crucian carp *Carassius carassius*. Mol Ecol.

[84] Henden L, Lee S, Mueller I, Barry A, Bahlo M (2018). Identity by descent analyses for measuring population dynamics and selection in recombining pathogens. PLoS Genet.

[85] Taylor AR, Schaffner SF, Cerqueira GC, Nkhoma SC, Anderson TJC, Sriprawat K (2017). Quantifying connectivity between local *Plasmodium falciparum* malaria parasite populations using identity by descent. PLoS Genet.

[86] Hartl DL, Clark AG (2006). Principles of Population Genetics.

[87] Taylor AR, Jacob PE, Neafsey DE, Buckee CO (2019). Estimating relatedness between malaria parasites. Genetics.

