# Supplementary material for: SNP barcodes provide higher resolution than microsatellite markers to measure Plasmodium vivax population genetics
Source: Malar J. 2020 Oct 20;19:375. doi: 10.1186/s12936-020-03440-0 (PMC7576724; doi:10.1186/s12936-020-03440-0)
Supplement: Supplementary file 2 — Additional file 2: Figure S1. Overview of steps used to select informative SNPs from P. vivax whole genome sequence data. Figure S2. Overview of amplicon sequencing approach for multiple samples (96) to amplify all target SNP loci (178) in single sequencing run. Genomic loci were amplified using standard PCR (PCR#1) using locus specific primer with universal overhang adaptors (a). Using purified primary PCR product as a template an additional PCR, index PCR (PCR#2) (b) was performed to attach a multiplex identifier (MID) tag which is unique to each sample and attaches to universal overhang sequence. Unique sequence of MID for each sample enables pooling of secondary PCR products (96 samples in this protocol) for library preparation (c). Then high throughput “multiplexed” sequencing of the combined amplicons from all samples (96) in a single MiSeq run to produce require millions of paired-end reads (2x300bp) (d). e Data was analysed using standard bioinformatics tools and mapped reads were visualized using Integrative Genomics Viewer. The graph shows a large number of reads covering the target SNP locus. This result shows that all reads possess the alternative allele (green) at the SNP site. This high frequency of SNP calls amongst reads gives high confidence to differentiate true SNPs (indicated as SNPS at target locus) from sequencing artefacts (rare SNPs shown left and right side of target locus). Figure S3. Overview of bioinformatic data analysis pipeline. Data processing sequence read consisting of quality checking of raw sequence reads, primers and adaptor trimming, mapping reads to reference sequence, SNP calling and filtering, and population genetic and statistical analyses. Figure S4. Quality control of the Plasmodium vivax barcoding assay. a Assessment of amplification bias and SNP polymorphism among genotyped samples. Comparison of number of successfully amplified loci before and after rWGA of samples. There was no statistically significant difference in n [file 12936_2020_3440_MOESM2_ESM.pptx]

## Slide 1
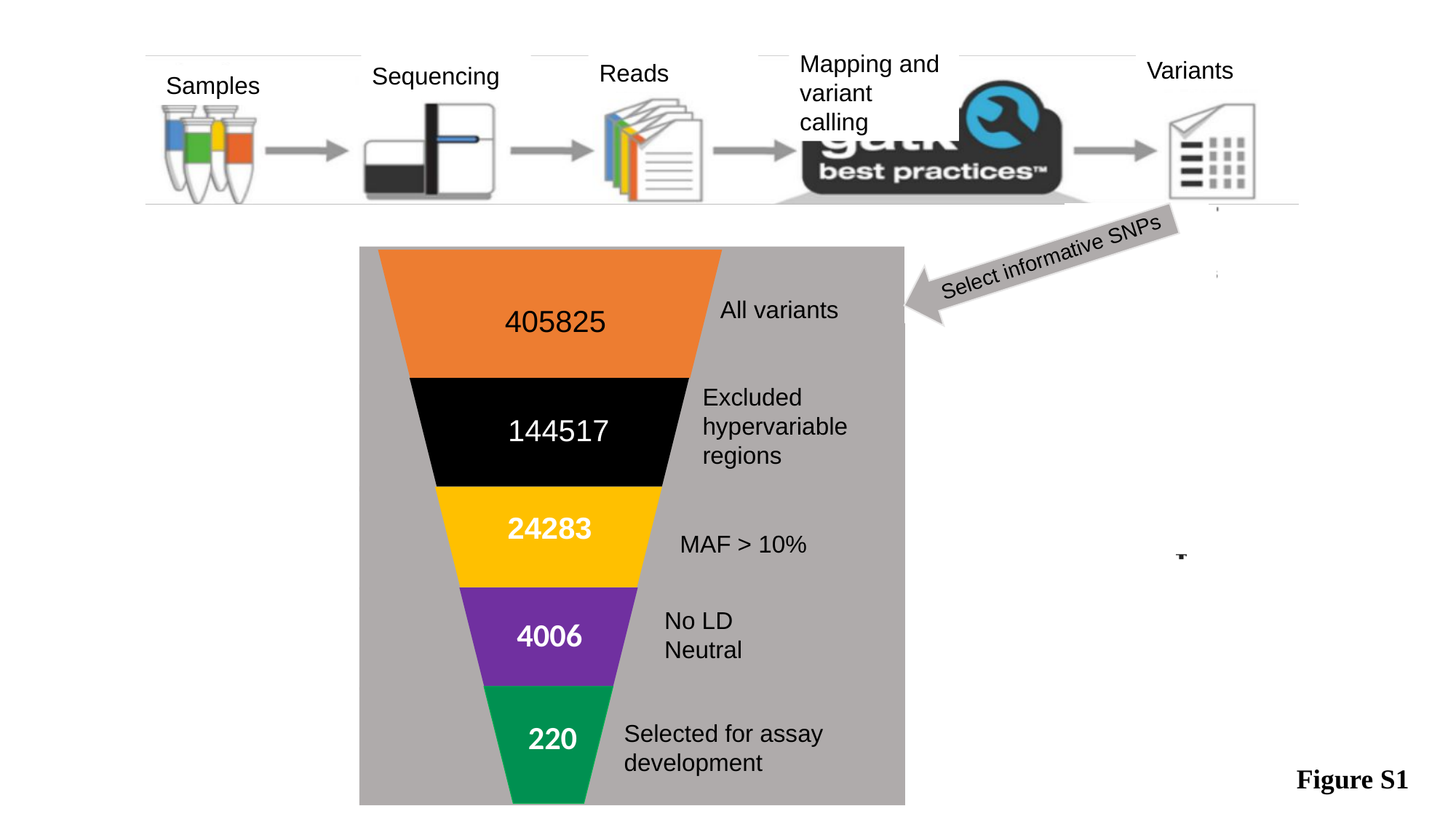

Mapping and variant calling
Variants
Reads
Sequencing
Samples
Select informative SNPs
 All variants
405825
Excluded hypervariable regions
144517
24283
MAF > 10%
No LD
Neutral
4006
220
Selected for assay development
Figure S1

## Slide 2
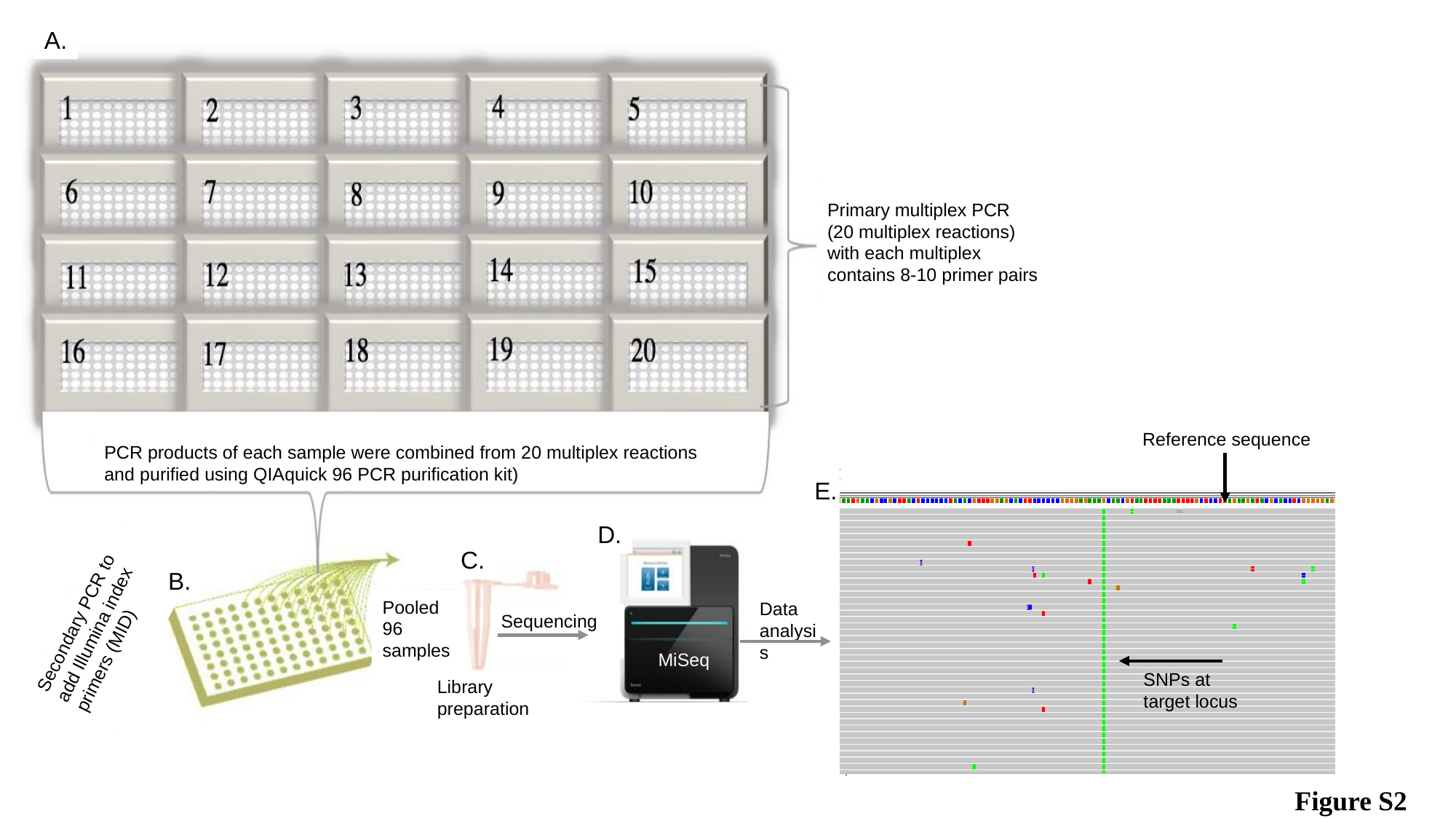

A.
Primary multiplex PCR
(20 multiplex reactions)
with each multiplex contains 8-10 primer pairs
PCR products of each sample were combined from 20 multiplex reactions and purified using QIAquick 96 PCR purification kit)
Reference sequence
SNPs at
target locus
E.
D.
C.
B.
Data
analysis
Secondary PCR to add Illumina index primers (MID)
Pooled 96 samples
Sequencing
MiSeq
Library preparation
Figure S2

## Slide 3
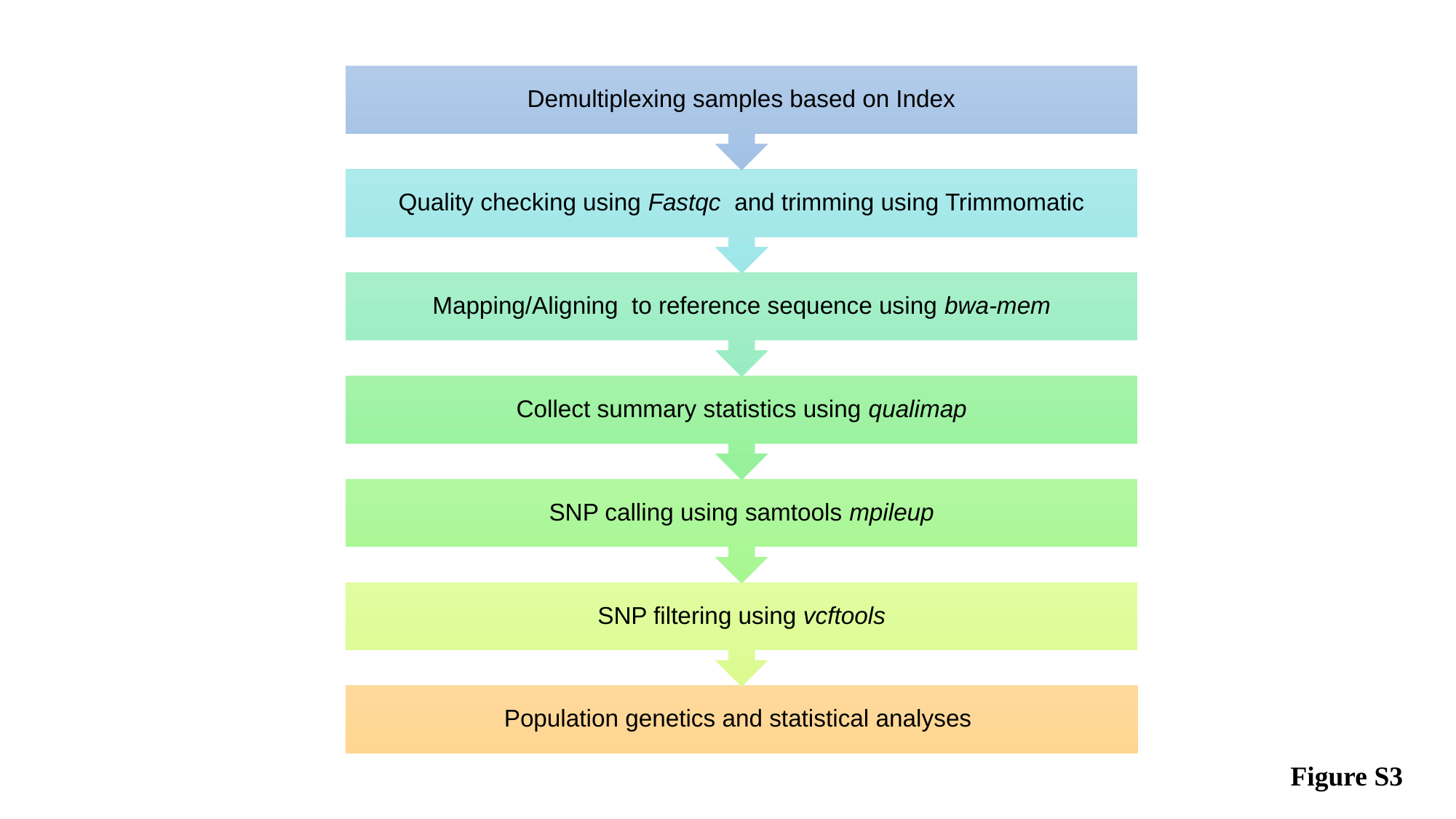

Figure S3

## Slide 4
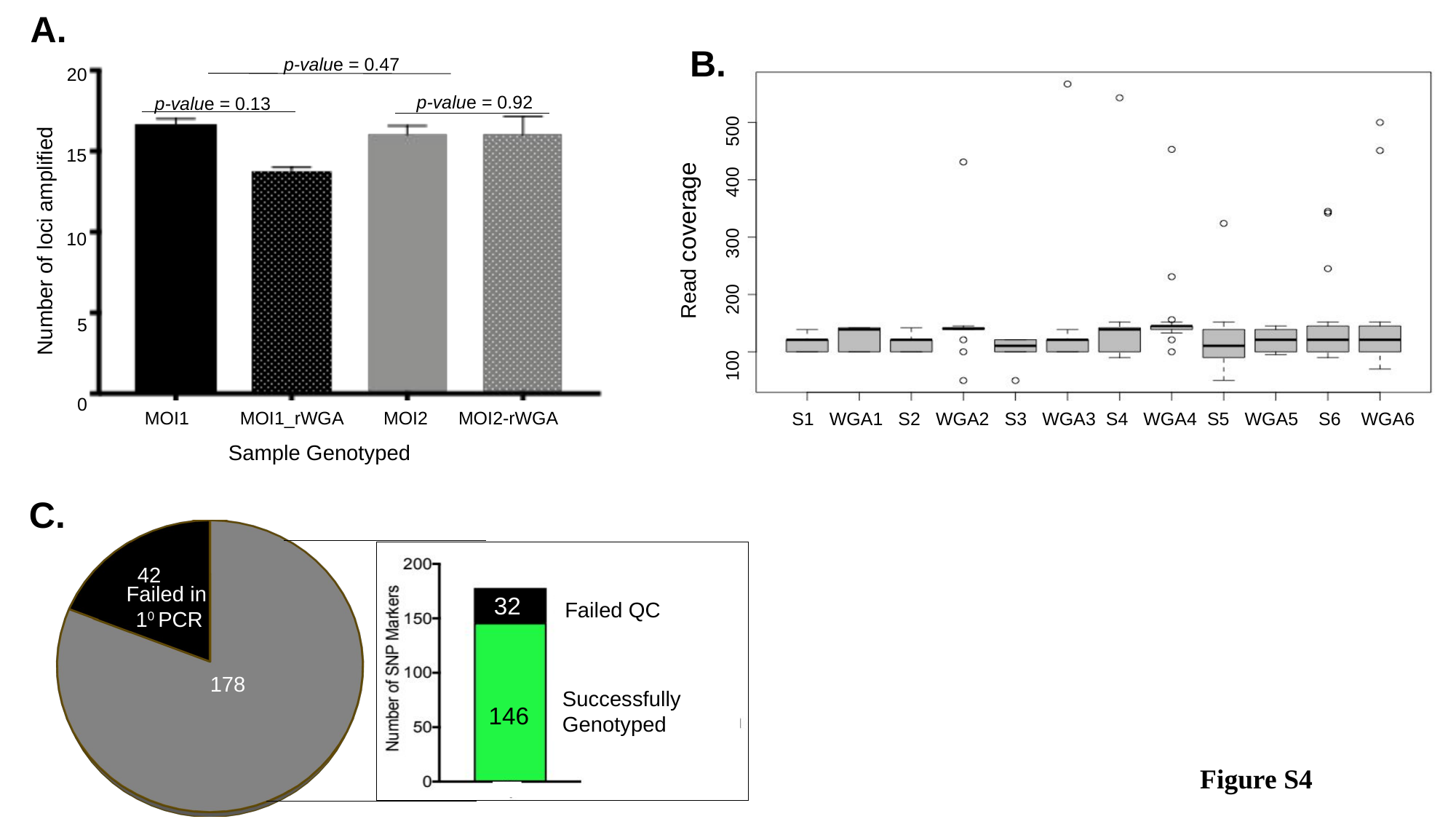

A.
p-value = 0.47
20
p-value = 0.92
p-value = 0.13
15
Number of loci amplified
10
5
0
 MOI1 MOI1_rWGA MOI2 MOI2-rWGA
Sample Genotyped
B.
Read coverage
100 200 300 400 500
 S1 WGA1 S2 WGA2 S3 WGA3 S4 WGA4 S5 WGA5 S6 WGA6
C.
[unsupported chart]
42
Failed in
 10 PCR
32
Failed QC
178
Successfully Genotyped
146
Figure S4

## Slide 5
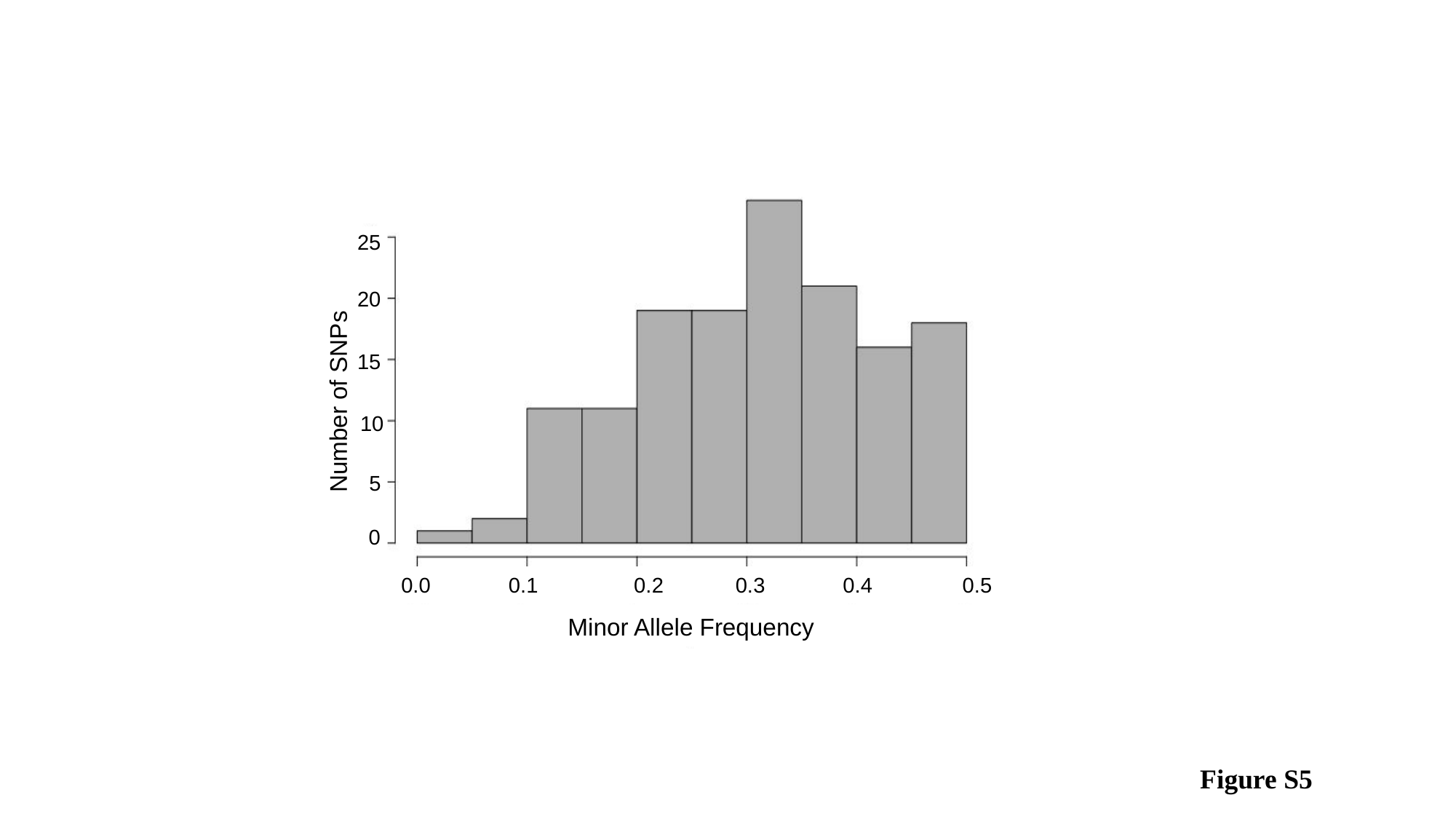

25
20
Number of SNPs
15
10
5
0
0.0 0.1 0.2 0.3 0.4 0.5
Minor Allele Frequency
Figure S5

## Slide 6
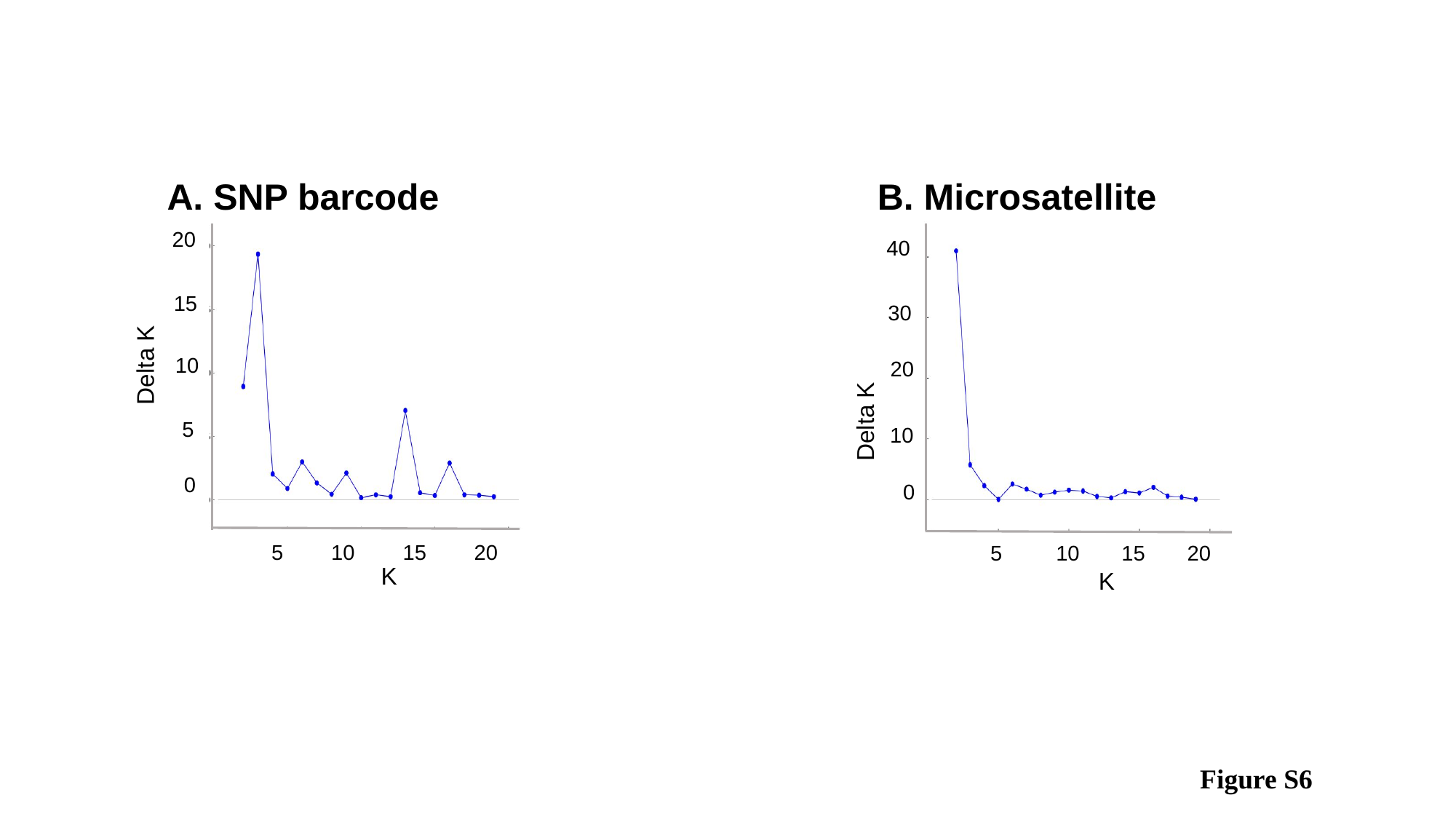

A. SNP barcode
B. Microsatellite
20
15
10
5
0
 5 10 15 20
K
40
30
20
10
0
Delta K
Delta K
 5 10 15 20
K
Figure S6

## Slide 7
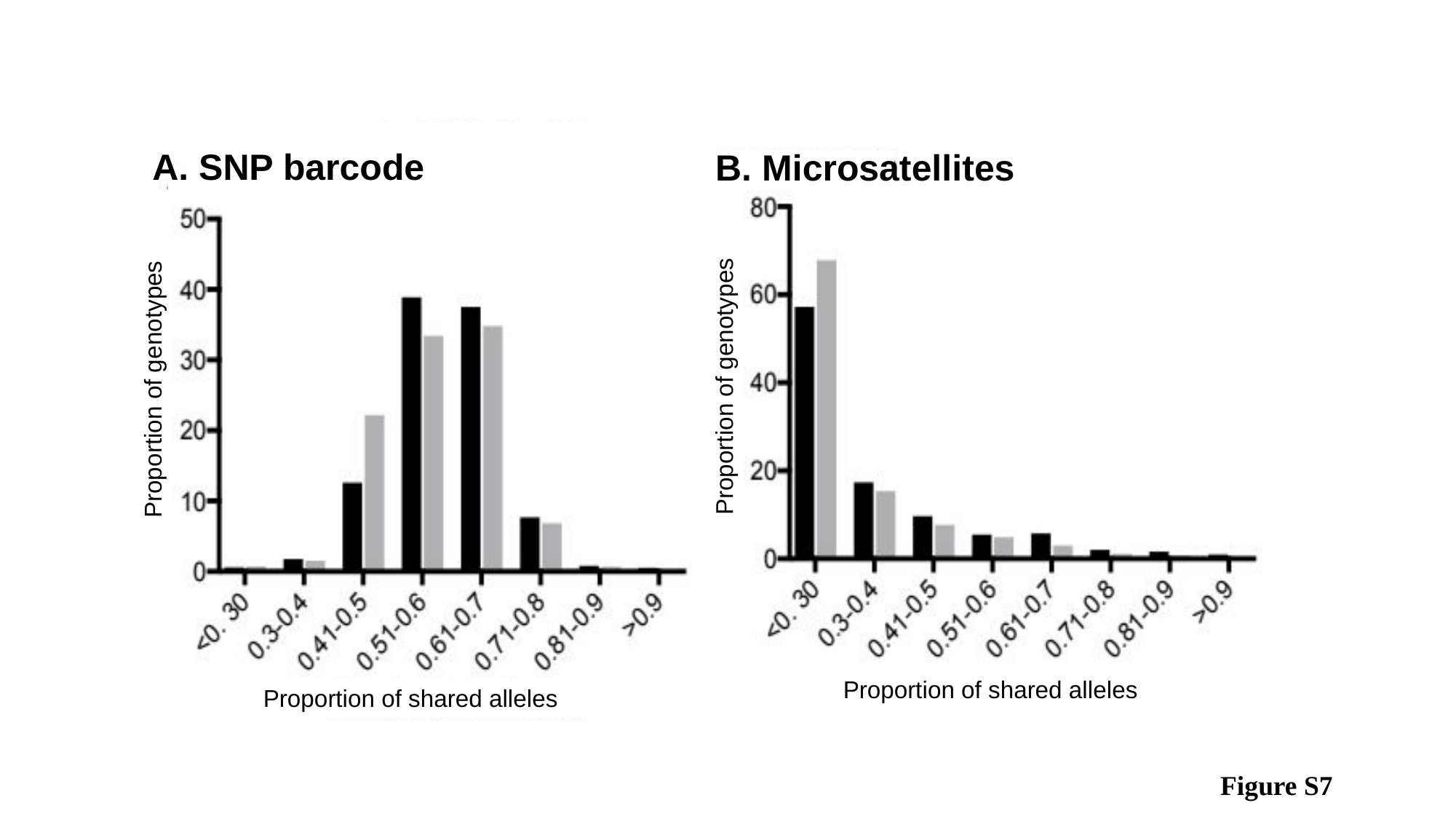

B. Microsatellites
Proportion of genotypes
Proportion of genotypes
Proportion of shared alleles
Proportion of shared alleles
A. SNP barcode
Figure S7

## Slide 8
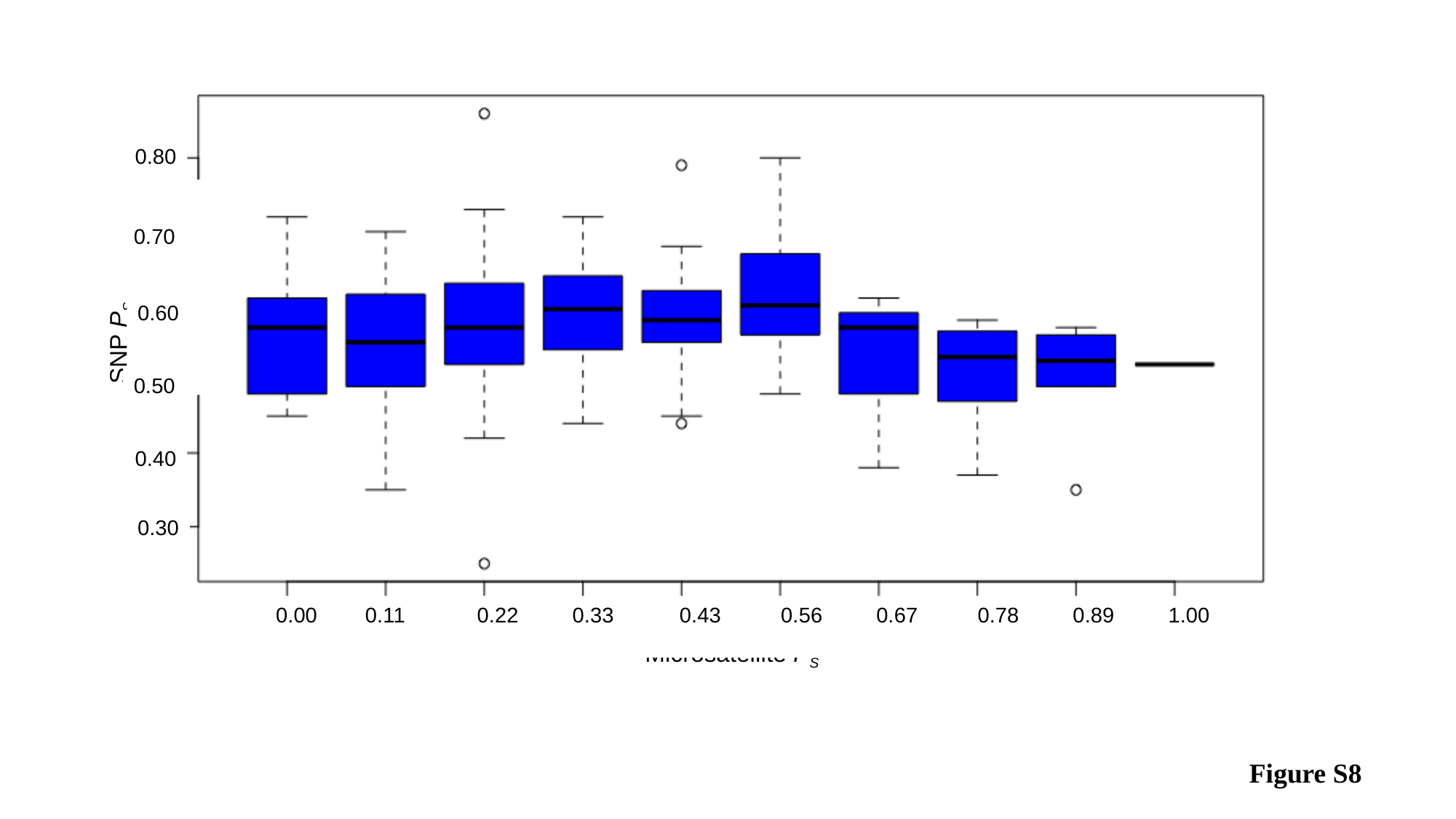

0.80
0.70
SNP PS
0.60
0.50
0.40
0.30
0.00 0.11 0.22 0.33 0.43 0.56 0.67 0.78 0.89 1.00
Microsatellite PS
Figure S8
